# Hierarchical Spatiotemporal Electroencephalogram Feature Learning and Emotion Recognition With Attention-Based Antagonism Neural Network

**DOI:** 10.3389/fnins.2021.738167

**Published:** 2021-12-02

**Authors:** Pengwei Zhang, Chongdan Min, Kangjia Zhang, Wen Xue, Jingxia Chen

**Affiliations:** School of Electronic Information and Artificial Intelligence, Shaanxi University of Science and Technology, Xi’an, China

**Keywords:** EEG, emotion recognition, spatiotemporal features, attention, antagonism neural network, BiLSTM

## Abstract

Inspired by the neuroscience research results that the human brain can produce dynamic responses to different emotions, a new electroencephalogram (EEG)-based human emotion classification model was proposed, named R2G-ST-BiLSTM, which uses a hierarchical neural network model to learn more discriminative spatiotemporal EEG features from local to global brain regions. First, the bidirectional long- and short-term memory (BiLSTM) network is used to obtain the internal spatial relationship of EEG signals on different channels within and between regions of the brain. Considering the different effects of various cerebral regions on emotions, the regional attention mechanism is introduced in the R2G-ST-BiLSTM model to determine the weight of different brain regions, which could enhance or weaken the contribution of each brain area to emotion recognition. Then a hierarchical BiLSTM network is again used to learn the spatiotemporal EEG features from regional to global brain areas, which are then input into an emotion classifier. Especially, we introduce a domain discriminator to work together with the classifier to reduce the domain offset between the training and testing data. Finally, we make experiments on the EEG data of the DEAP and SEED datasets to test and compare the performance of the models. It is proven that our method achieves higher accuracy than those of the state-of-the-art methods. Our method provides a good way to develop affective brain–computer interface applications.

## Introduction

Emotion plays an important role in human life (Picard and Picard, 1997). Positive emotions may help improve the efficiency of our daily work, while negative emotions may affect our decision making, attention, and even health (Picard and Picard, 1997). Although it is easier for us to recognize emotions of other people from their facial expression or voice, it is still difficult for machines to do that (Li et al., 2019). In the past few years, emotion recognition by computer has attracted more and more researchers, and it has become a hot research topic in the field of affective computing and pattern recognition (Purnamasari et al., 2017). The emotion recognition methods can be based on speech signals, facial expression images, and physiological signals (Chen et al., 2019a). In recent years, EEG-based emotion recognition algorithms have been increasingly focused on by researchers.

While researching emotion recognition with EEG, we usually face two difficulties. One is how to obtain a discriminative feature representation method from original EEG signals, and the other is how to build an effective model to better improve the performance of emotion classification. Technically, EEG features can be extracted from the time domain, frequency domain, and time–frequency domain (Jenke et al., 2014). For example, Zhang and Lee (2010) regarded the amplitude difference of symmetric electrodes in the time domain as the EEG feature of emotion recognition. Lin et al. (2010) studied the relationship between emotional state and brain activity, and extracted power spectral density, differential asymmetric power, and reasonable asymmetric power separately as features of EEG signals. Duan et al. (2013) extracted features by calculating the correlation coefficient between the features of each frequency band and their emotional labels. In the aspect of models, Garcia-Martinez et al. (2019) summarized the research results of applying nonlinear methods to EEG signal analysis in recent years. Li et al. (2018b) proposed a graph-regularized sparse linear regression model to make emotion classification and achieved better recognition performance. Zheng and Lu (2015) studied the key frequency bands and key brain regions of EEG signals, and proposed to use group sparse canonical correlation analysis algorithm (Zheng, 2017) for multichannel EEG-based emotion recognition.

With the development of artificial intelligence, deep learning has become very popular, and emotion classification based on deep learning has also continuously improved the performance of emotion recognition and, thus, has gradually become the dominant method. Alhagry et al. (2017) proposed an end-to-end LSTM-RNN network to learn the time dependence of EEG signals. Li et al. (2018a) considered the area shift of EEG data and used deep neural network to learn the difference between left and right hemispheres to narrow the distribution shift. Song et al. (2018) established a graph relationship based on multichannel EEG data, adjacency matrix to build the internal relationship between different EEG channels, and then used dynamical graph convolution network to extract features for emotion classification. Salama et al. (2018) used a three-dimensional convolutional neural network (3D-CNN) to recognize emotions from multichannel EEG data. The author of this paper has also proposed a deep CNN model (Chen et al., 2019c) to learn high-level discriminative feature representations from the combined features of the EEG signal in the time-frequency domain. In Chen et al. (2019b), a hierarchical bidirectional LSTM model based on attention mechanism was proposed to reduce the influence of long-term instability of EEG sequences on emotion recognition.

Although many EEG emotion recognition methods have emerged recently, there are still some problems that needs to be further studied. One of the problems is how to obtain effective high-level features from the original EEG signals automatically. Most researchers often extract some time or frequency statistical EEG features manually combined with classic machine learning algorithms to make emotion classification. However, feature engineering needs to consume a lot of computation resources and time. It is expected to automatically learn more prominent spatiotemporal features with less feature engineering. The second question is which brain area contributes more to human emotion recognition, and how to use the distribution information of different brain areas to improve recognition performance. The latest researches (Etkin et al., 2011; Lindquist and Barrett, 2012) have shown that human emotions are closely related to multiple areas of the cerebral cortex, such as the orbitofrontal cortex, ventromedial prefrontal cortex, amygdala, and so on. Therefore, the contribution of EEG signals associated with each brain area is different. If the spatial information of different brain regions can be used, it is expected to provide help in understanding human emotions (Heller and Nitscke, 1997; Davidson, 2000; Lindquist et al., 2012). The third question is how to enhance the emotion recognition performance by using time series information in each brain area, as EEG signals are dynamic time series carrying important emotion dynamics, which is effective to identify human emotions.

Literature (Lin et al., 2010; Zhang and Lee, 2010; Duan et al., 2013; Zheng and Lu, 2015; Zheng, 2017; Li et al., 2018b; Garcia-Martinez et al., 2019) has proven that EEG signals in different brain regions have different contributions to emotion recognition. Literature (Alhagry et al., 2017; Li et al., 2018a; Salama et al., 2018; Song et al., 2018; Chen et al., 2019b) found that either deep CNN model or the bidirectional long- and short-term memory (BiLSTM) model combined with attention mechanism could hierarchically extract deep temporal and spatial context of EEG signals. Inspired by these two aspects and neuroscience research basis (Heller and Nitscke, 1997; Davidson, 2000; Etkin et al., 2011; Lindquist and Barrett, 2012; Lindquist et al., 2012), this paper proposes a new emotion computing model called R2G-ST-BiLSTM, which is used to solve the above three main problems. Its core idea is to extract the EEG spatial temporal dynamics associated with human emotions from local and global brain areas. Specifically, the R2G-ST-BiLSTM model contains two two-layer neural networks, in space and time domain, respectively, and features are learned hierarchically from region to global (R2G) to grasp more discriminative spatiotemporal EEG features related to human emotions. The proposed R2G-ST-BiLSTM model consists of three parts:

(1) Feature learning module. It uses the bidirectional long- and short-term memory (BiLSTM) network to learn the hierarchical spatiotemporal EEG characteristics within and between each brain region. In order to better judge the effect of different brain regions on emotion recognition, this paper introduces the regional attention mechanism to learn a set of weights, which represent the contributions of different brain regions.
(2) Emotion classifier. The purpose of this module is to predict emotion category based on EEG spatiotemporal features obtained by feature learning module. At the same time, it also guides the whole neural network to learn more discriminative EEG features for emotion classification.
(3) Domain discriminator. This module aims to decrease the domain offset between the training EEG data and the testing EEG data through introducing a discriminator, so that the hierarchical feature learning module can produce EEG features with more emotional discrimination and stronger domain adaptability.

Through collaborative work of the above three modules, the R2G-ST-BiLSTM model can learn EEG features with better discrimination ability and domain robustness simultaneously, thus, further improving human emotion recognition performance. Overall, there are three main contributions in our work:

• Inspired by neuroscience, we propose a new hierarchical spatiotemporal EEG feature learning model, which obtains spatiotemporal emotional information from EEG data within and between each cerebral region.
• Proposes an attention weighted model to estimate the contribution of each cerebral region to the different affections of humans. The influence of the most dependent cerebral region is enhanced by the learned weight, and the impact of the less dependent region was reduced as well.
• Proposes a domain discriminator to work on antagonism with the classifier to improve the adaptability of the R2G-ST-BiLSTM model.

## Method Based on the R2G-ST-BiLSTM Model

Traditional one-way LSTM network (Hochreiter and Schmidhuber, 1997) has a special structure that is different from the simple recurrent neural network (RNN) (Graves et al., 2013) and is more capable of dealing with the frequent dependence of the sample sequence. Its special “gate” structures enable LSTM to retain significant data information and forget unnecessary redundant information (Yan et al., 2017). However, one disadvantage of this network is that it only uses the context-related information that happened before. The BiLSTM network can process data by using separate hidden layers in two directions (Bottou, 2010). Because the BiLSTM network can obtain long-term contextual information in both forward and backward orientation, it is better than the traditional one-way LSTM network for modeling time series. Because EEG data related to each channel in each brain region are in time series with the same dimension, therefore, BiLSTM can be used to extract the deep spatiotemporal context features of EEG data from the local brain regions to the global brain.

**FIGURE 1:**
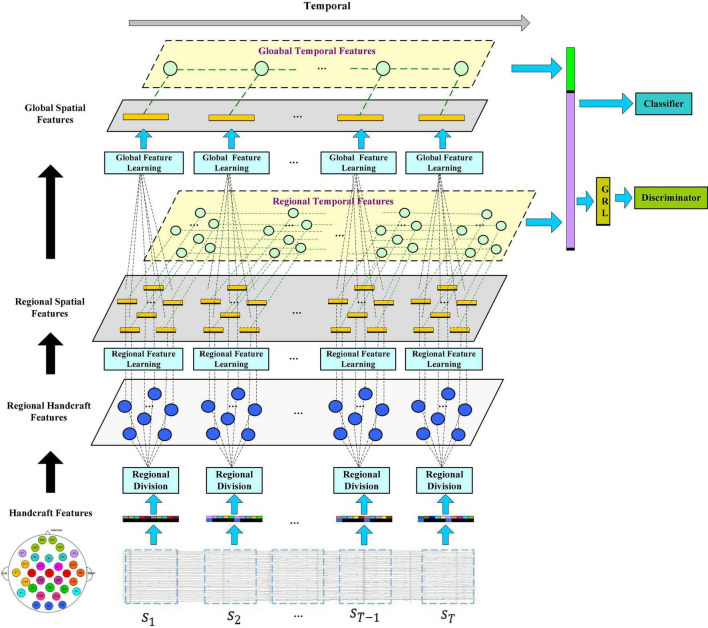
The model of the R2G-ST-BiLSTM network. Feature learning is processing from regional brain to global brain, respectively, in the spatial and temporal flows. Spatial flow learns the relationship between brain regions in different layers, while temporal flow learns the emotion-related EEG dynamic from the time series of each brain region.

In this section, we will introduce the framework of the R2G-ST-BiLSTM model in detail and explain the specific application of EEG signals for emotion recognition methods and procedures. Figure 1 shows the framework of the R2G-ST-BiLSTM model. It consists of three main modules, which are feature extractors, classifiers, and discriminators.

### Spatial Feature Extraction

First, we divide the EEG sequence into several equal-length segments. Then a set of manual features is extracted from the EEG segments corresponding to each electrode. For example, the differential entropy feature (DE feature) is extracted from δ(1∼4 hz), θ(5∼8 hz), α(9∼14 hz), β(15∼3 0hz), and γ(31∼50 hz) (Zheng and Lu, 2015). In addition, to capture dynamic time information from input EEG sequence, every five adjacent EEG segments make up one EEG sample, and each EEG sample is represented by a tensor of its manual feature.

Let *S* = [*s*_1_, *s*_2_, *s*_*T*−1_, *s*_*T*_]ϵℝ^*d* × *n* × *T*^ represent an EEG sample, where *s_i_* represents the feature data extracted from the divided *i*-th segment of EEG, shown in the bottom blue rectangle of Figure 1, *d* is the number of EEG features per channel, *n* is the number of channels, and *T* is the number of segments per EEG sample. Figure 1 shows that when extracting spatial features, each sample includes a regional feature extraction layer and a global feature extraction layer to gradually learn high-level semantic features from local to global.

**FIGURE 2:**
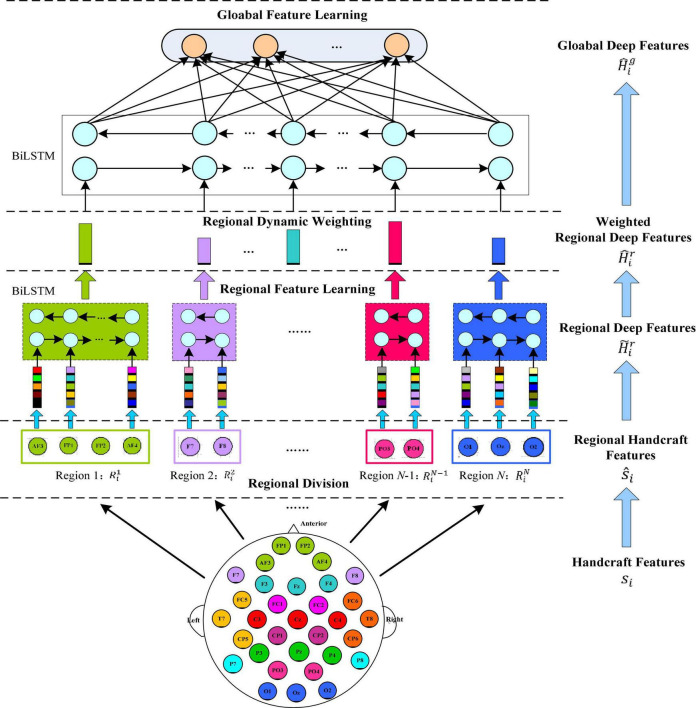
Regional to global spatial feature learning process consisting of regional feature learning layer, dynamic weight layer, and global feature learning layer.

Figure 2 shows the specific feature learning process of the EEG data *s_i_*. At first, the channels of *s_i_* are grouped into different areas according to the spatial position of the brain electrodes. The number of electrodes in each brain area varies due to the different functions of each brain area, thereby generating a set of regional manual feature vectors in each brain region. Then these manual feature vectors are input into the equal number of BiLSTM networks to learn the local abstract features of each region. After learning the regional deep features, the region attention mechanism is introduced to learn a set of weights that represent the significance of each region. Finally, at the top of Figure 1, the extracted weighted feature of each region is input into another set of BiLSTM networks to further extract the global emotional semantic features.

(1) *Regional feature extraction layer.* Let *x*_*ij*_ represent the manual feature vector of the *j-*th EEG channel, so *s*_*i*_ = [*x*_*i*1_, *x*_*i*2_, …, *x*_*in*_]ϵℝ^*d*×*n*^. Then according to the related electrodes, *n* channels of *s_i_* are divided into different groups: each group of channels belongs to a cerebral area, and each area is expressed as: brain area 1: $R_{i}^{1}=\left[x_{i1}^{1},x_{i2}^{1},\ldots ,x_{in_{1}}^{1}\right],$brain area 2: $R_{i}^{2}=\left[x_{i1}^{2},x_{i2}^{2},\ldots ,x_{in_{2}}^{2}\right],$brain area n: $R_{i}^{N}=\left[x_{i1}^{N},x_{i2}^{N},\ldots ,x_{in_{N}}^{N}\right]$, where *N* is the quantity of cerebral areas, *n_j_* is the quantity of the *j-*th cerebral area of the channels, and *n*_1_ + + *n*_*N*_ = *n*. Furthermore, we adjust the column order of *s_i_*, which is represented as a new matrix ${\hat{s}}_{i}=\left[R_{i}^{1},\ldots ,R_{i}^{N}\right]$. The submatrix $R_{i}^{j}\left(j=1,\ldots ,N\right)$ represents cerebral area *j*, and per column of $R_{i}^{j}$ corresponds to an EEG channel of this area. The spatial relationship of the brain area can be modeled by a BiLSTM working on the $R_{i}^{j}$ matrix to extract the advanced features of each region, which process is expressed as:


(1) $$ℱ\left(R_{i}^{1}\right)=\left[{\tilde{h}}_{i1}^{1},{\tilde{h}}_{i2}^{1},\ldots ,{\tilde{h}}_{in_{1}}^{1}\right]\epsilon {\mathbb{R}}^{2d_{r}\times n_{1}},$$

(2) $$ℱ\left(R_{i}^{N}\right)=\left[{\tilde{h}}_{i1}^{N},{\tilde{h}}_{i2}^{N},\ldots ,{\tilde{h}}_{in_{N}}^{N}\right]\epsilon {\mathbb{R}}^{2d_{r}\times n_{N}},$$

where ℱ(⋅) represents the BiLSTM operation, ${\tilde{h}}_{ik}^{j}\epsilon {\mathbb{R}}^{2d_{r}}$ represents hidden vectors output by the *k*th forward and backward hidden units of the BiLSTM, and *d_r_* represents the dimensions of the output state vectors of each hidden unit in the BiLSTM. At last, the state vector outputs by the last hidden unit of each BiLSTM are connected as the local deep features of all regions, which are expressed as follows:


(3) $${\tilde{H}}_{i}^{r}=\left[{\tilde{h}}_{in_{1}}^{1},{\tilde{h}}_{in_{2}}^{1},\ldots ,{\tilde{h}}_{in_{N}}^{N}\right]\epsilon {\mathbb{R}}^{2d_{r}\times N}.$$

For simplicity, each BiLSTM model in this part is initialized and fit jointly, and the hyperparameters are shared with each other.

(2) *Attention-based brain region weighting layer.* Neuroscience-related research shows that different brain areas respond to different types of emotions. Therefore, EEG signals from diverse brain areas have different contributions to emotion classification. To emphasize the role of the different brain area electrodes in EEG emotion recognition, we introduce a weighting layer based on attention mechanism. Expressed by *W* = {*w*_*ij*_}, it can characterize the significance of channels in different areas. After that, the local deep features of all areas are expressed by ${\hat{H}}_{i}^{r}$ as follows:


(4) $${\hat{H}}_{i}^{r}={\tilde{H}}_{i}^{r}W,$$

(5) $$W={\left(U\tanh\left(V{\tilde{H}}_{i}^{r}+b^{r}e^{T}\right)\right)}^{T},$$

(6) $$w_{ij}=\frac{\text{exp}\left(w_{ij}\right)}{\sum_{k=1}^{N}\text{exp}\left(w_{ik}\right)},$$

where *U* and *V* are learnable transpositional matrices, *b^r^* represents the deviation, and *e* represents an N-dimensional vector whose elements are 1, that is, *e* = [1, 1, …, 1]^*T*^. The matrix *W* is normalized across the columns, so that its values are limited to non-negative by formula (6). The larger the *w*_*ij*_ value obtained, the more important the *j-*th brain area is for emotion recognition.

(3) *Global feature extraction layer.* To further capture the potential global structural information on the basis of the learned local deep feature ${\hat{H}}_{i}^{r}$, we use another BiLSTM network with *N* hidden units to extract global spatial features.


(7) $$ℱ\left({\hat{H}}_{i}^{r}\right)=\left[{\tilde{h}}_{i1}^{g},{\tilde{h}}_{i2}^{g},\ldots ,{\tilde{h}}_{iN}^{g}\right]\epsilon {\mathbb{R}}^{2d_{g}\times N},$$

where, ${\tilde{h}}_{ik}^{g}$ represents the hidden vector output by the *k-*th forward and backward hidden unit of the BiLSTM network, and *d_g_* is the dimension of the output state vector of each hidden unit. Next, input the vector sequence ${\tilde{h}}_{i1}^{g}$,…,${\tilde{h}}_{iN}^{g}$ into a fully connected layer to learn a new compressed feature vector with the following formulas:


(8) $${\hat{h}}_{jK}^{g}=\sigma \left(\sum_{j=1}^{N}P_{jk}^{g}{\tilde{h}}_{ij}^{g}+b^{g}\right),k=1,2,\ldots ,K,$$

(9) $${\hat{H}}_{i}^{g}=\left[{\hat{h}}_{i1}^{g},{\hat{h}}_{i2}^{g},\ldots ,{\hat{h}}_{iK}^{g}\right],$$

where $P^{g}={\left[P_{jK}^{g}\right]}_{N=\times K}$ denotes a projection matrix, *b^g^* denotes deviation, *K* denotes the length of the compressed sequence, and σ(⋅) is a nonlinear function. Thus, the global deep feature ${\hat{H}}_{i}^{g}$ related to the manual feature matrix *s_i_* of the *i-*th EEG segment is finally obtained.

### Temporal Feature Extraction

Let ${\tilde{h}}_{i}^{j}\left(j=1,\ldots ,N\right)$ represent the state vector output by the *j*-th brain region of the *i*-th EEG manual feature matrix *s_i_* through the last hidden unit of the BiLSTM network, then the time series of each brain region feature can be expressed as:


(10) $${\tilde{H}}^{r1}≜\left[{\tilde{h}}_{1}^{1},{\tilde{h}}_{2}^{1},\ldots ,{\tilde{h}}_{T}^{1}\right],$$

(11) $${\tilde{H}}^{rN}≜\left[{\tilde{h}}_{1}^{N},{\tilde{h}}_{2}^{N},\ldots ,{\tilde{h}}_{T}^{N}\right]$$

In this way, the columns of the feature matrix ${\tilde{H}}^{rj}\left(j=1,\ldots ,N\right)$ constitute the time series of the feature vectors related to the *j*-th brain region. Therefore, a BiLSTM network can be applied again to learn the temporal context between these eigenvector sequence:


(12) $$\begin{array}{c} Z^{rt}=\left[ℱ\left({\tilde{H}}^{r1}\right),\ldots ,ℱ\left({\tilde{H}}^{rN}\right)\right]=\left[\left(z_{11}^{rt},\ldots ,z_{1T}^{rt}\right),\ldots ,\left(z_{N1}^{rt},\ldots ,z_{NT}^{rt}\right)\right]\\ \;=\left[\left(z_{1}^{rt},\ldots ,z_{N}^{rt}\right)\right], \end{array}$$

where $Z_{j}^{rt}=\left[z_{j1}^{rt},\ldots ,z_{jT}^{rt}\right]\epsilon {\mathbb{R}}^{2d_{rt}=T}$ represents the regional temporal feature matrix related to the *j*-th brain region, and *d*_*rt*_ is the dimension of each hidden unit state vector in the regional temporal BiLSTM network. Take the output $z_{jT}^{rt}$ of the last hidden unit of the BiLSTM network in each brain area as the learned temporal feature of this brain area, and then get the final temporal feature *z^rt^* of all brain areas, which is expressed as:


(13) $$z^{rt}=\left[{\left(z_{1T}^{rt}\right)}^{T},{\left(z_{2T}^{rt}\right)}^{T},\ldots ,{\left(z_{NT}^{rt}\right)}^{T}\right].$$

In addition, to explore the time context on the basis of matrix ${\hat{H}}_{i}^{g}$, we convert the columns of ${\hat{H}}_{i}^{g}$ to a new sequence, which is represented by ${\hat{h}}_{i}^{g}$:


(14) $${\hat{h}}_{i}^{g}={\left[{\left({\hat{h}}_{i1}^{g}\right)}^{T},{\left({\hat{h}}_{i2}^{g}\right)}^{T},\ldots ,{\left({\hat{h}}_{iK}^{g}\right)}^{T}\right]}^{T}.$$

Set up $Z^{g}=\left[{\hat{h}}_{1}^{g},\ldots ,{\hat{h}}_{T}^{g}\right]$. Then a BiLSTM network with *T* hidden units is used to learn the global temporal feature *Z^gt^*:


(15) $$Z^{gt}=ℱ\left(Z^{g}\right)=\left[z_{1}^{gt},\ldots ,z_{T}^{gt}\right]\epsilon {\mathbb{R}}^{2d_{gt}\times T},$$

where *d*_*gt*_ denotes the size of the hidden state vector of the global temporal BiLSTM network, and the output $z_{T}^{gt}$ of the last hidden unit is taken as the learned global temporal feature. Finally, by concatenating *z^rt^* with $z_{T}^{gt}$, the optimal feature vector *z^rg^* of the EEG sample *S* (composed of *T* EEG fragments) is obtained, which contains complex temporal context information, and its expression is:


(16) $$z^{rg}={\left[{\left(z_{1T}^{rt}\right)}^{T},{\left(z_{2T}^{rt}\right)}^{T},\ldots ,{\left(z_{NT}^{rt}\right)}^{T},{\left(z_{T}^{gt}\right)}^{T}\right]}^{T}.$$

### Classifier and Discriminator

For the final eigenvector *z^rg^* input to this layer, a simple linear transformation method can be used to recognize the human emotional type of the input EEG data *S* as the following formula:


(17) $$Y=Qz^{rg}+b_{c}=\left[y_{1},y_{2},\ldots ,y_{c}\right],$$

where *Q* and *b_c_*, respectively, denotes the projection matrix and deviation. *C* is number of emotional categories. The element of the transformation result *Y* is input into a softmax function to predict the emotion category:


(18) $$P\left(c|S\right)=\max\left\{\frac{\exp\left(y_{k}\right)}{\sum_{i=1}^{C}\exp\left(y_{i}\right)}|k=1,\ldots ,C\right\},$$

where *P*(*c*|*X*) represents the probability the input EEG data *S* is predicted to be the emotion of type *c*.

Supposing the training set of the model is composed of *M* EEG data, which is expressed by matrix $S_{i}^{S}\left(i=1,\ldots ,M\right)$. The loss function of the emotion classifier can be expressed as:


(19) $${ℒ}_{c}\left(S_{1}^{S}\ldots ,S_{M}^{S};\theta_{f},\theta_{c}\right)=\sum_{i=1}^{M}\sum_{c=1}^{C}-\varphi \left(l_{i},c\right)\times \log P\left(c|S_{i}^{S}\right)$$

where *l_i_* represents the real label of the $S_{i}^{S}$ sample, and θ_*f*_ and θ_*c*_ represent the learning parameters. φ(*l*_*i*_, *c*) is expressed as:


(20) $$\varphi \left(l_{i},c\right)=\left\{ \begin{array}{c} 1,ifl_{i}=c,\\ 0,otherwise. \end{array}\right.$$

From formulas (19) and (20), it can be concluded that by minimizing the loss function ${ℒ}_{c}\left(S_{1}^{S},S_{2}^{S},\ldots ,S_{M}^{S};\theta_{f},\theta_{c}\right)$, the emotion category of each training sample can be correctly predicted to the maximum extent.

Let *S*_*test*_ represent a test sample, and the emotion label of *S*_*test*_ is determined by the formula:


(21) $$l_{test}=arg\max_{c}\left\{P\left(c|S_{test}\right)|c=1,\ldots ,C\right\},$$

where *l*_*test*_ represents the predicted label of the test sample *S*_*test*_.

When performing prediction, the EEG samples for the training and testing data may be from various subjects and even different experiments. Based on this, the recognition model learned by using the training data may not have a high recognition accuracy for the test data. To optimize the generalization ability of the model, a discriminator is introduced to work collaboratively with the classifier to learn features with strong emotion discrimination and domain invariance.

Specifically, suppose that $D^{S}=\left\{S_{1}^{S},\ldots ,S_{M_{1}}^{S}\right\}$ denotes the dataset of the source domain, and $D^{T}=\left\{S_{1}^{T},\ldots ,S_{M_{2}}^{T}\right\}$ denotes the dataset of the target domain, where *M*_1_ and *M*_2_ are their sample number. To alleviate the domain difference, the loss function of the discriminator is defined as:


(22) $${ℒ}_{d}\left(S_{i}^{S},S_{j}^{T};\theta_{f},\theta_{d}\right)=-\sum_{i=1}^{M_{1}}logP\left(0|S_{i}^{S}\right)-\sum_{j=1}^{M_{2}}logP\left(1|S_{j}^{T}\right).$$

Here, $P\left(0|S_{i}^{S}\right)$ is the probability that EEG sample $S_{i}^{S}$ is classified into the source domain, $P\left(1|S_{j}^{T}\right)$ is the probability that EEG sample $S_{j}^{T}$ is classified into the target domain, and θ_*d*_ is the parameter. The discriminator enables this model to learn the domain-invariant features gradually.

### Optimization of the Bidirectional Long- and Short-Term Memory Neural Network From Region to Global Brain Model

The previous description indicates that through minimizing formula (19) and maximizing formula (22), domain difference can be reduced and better domain invariant characteristics can be learned. Therefore, we redefine the total loss function of R2G-ST-BiLSTM model as:


(23) $$ℒ\left(S^{S},S^{T}|\theta_{f},\theta_{c},\theta_{d}\right)={ℒ}_{c}\left(S^{S};\theta_{f},\theta_{c}\right)-{ℒ}_{d}\left(S^{S},S^{T};\theta_{f},\theta_{d}\right).$$

To optimize our model, we need to find the best parameters that minimize the new loss function ℒ(*S^S^*, *S^T^*|θ_*f*_, θ_*c*_, θ_*d*_). By minimizing ℒ_*c*_(*S^S^*; θ_*f*_, θ_*c*_) and maximizing ℒ_*d*_(*S^S^*, *S^T^*; θ_*f*_, θ_*d*_) synchronously and iteratively, the optimal parameters of ℒ(*S^S^*, *S^T^*|θ_*f*_, θ_*c*_, θ_*d*_) can be obtained. Specifically, the stochastic gradient descent (SGD) algorithm (Yu et al., 2015) is used to find the optimal model parameters:


(24) $$\left({\hat{\theta }}_{f},{\hat{\theta }}_{c}\right)|=arg\min_{\theta_{f},\theta_{c}}{ℒ}_{c}\left(S^{S},\left(\theta_{f},\theta_{c}\right),{\hat{\theta }}_{d}\right),$$

(25) $${\hat{\theta }}_{d}=arg\max_{\theta_{d}}{ℒ}_{d}\left(S^{S},S^{T}\left({\hat{\theta }}_{f},{\hat{\theta }}_{c}\right),\theta_{d}\right),$$

The feature extractor can learn to obtain emotional discriminative features by minimizing the loss function ℒ_*c*_. Meanwhile, it extracts domain invariant features by maximizing the loss function ℒ_*d*_. When obtaining the optimal parameters of the R2G-ST-BiLSTM model, we also introduced a gradient reverse layer (GRL) (Ganin et al., 2016), which performs gradient sign reversal when performing backward propagation operation and enables the discriminator to transform the maximization problem into a minimization problem, so that SGD can be used for parameter optimization. The parameter updating can be expressed as:


(26) $$\theta_{d}\leftarrow \theta_{d}-\alpha \frac{\partial {ℒ}_{d}}{\partial \theta_{d}},\theta_{f}\leftarrow \theta_{f}+\alpha \frac{\partial {ℒ}_{d}}{\partial \theta_{f}},$$

where α is the learning rate.

### Configuration and Training of Bidirectional Long- and Short-Term Memory Neural Network From Region to Global Brain Model

The proposed model is implemented in TensorFlow framework on a NVIDIA Titan × Pascal GPU-equipped work station and trained from scratch in a fully supervised manner. When training the whole model, we define a search space to find the optimal model parameters. The search space includes the hidden_layers (one to three layers), hidden_size (32, 64, 128, and 256), batch_size (30, 60, 80, and 120), learning_rate (0.1, 0.01, 0.001, and 0.0001), dropout (0.5, 0.6, and 0.7), and epochs (100, 200, 300, and 500). The search space was defined to balance the trade-off between a deeper architecture and limited training samples. For simplicity, each BiLSTM model is initialized and fit jointly, their hyperparameters are shared with each other, the hidden_size of the single-layer perception network used to learn the attention weight of each brain region is 128, the hidden_size of the full connection layer for learning the compressed global brain feature is 64, all hidden layers use ReLU activation function for faster approximation, all BiLSTM models are trained using SGD with AdaGrad optimizer, and the maximum training iteration was set to be 10,000. For searching each hyper parameter, we only adjust one hyperparameter in a defined search space and fix others each time. When observing that there is no growth trend of the accuracy on training and validation sets, we can judge to stop the training process in advance, as shown in Figures 4, 6. Finally, we select the best model that produces the highest accuracy on the validation dataset.

Through this fine-tuning process, the selected best hidden_layers is 2, the hidden_size of *d_r_*, *d_g_*, *d*_*rt*_, and *d*_*gt*_ is consistently 64, learning rate is 0.001, batch-size is 120, and epochs is 200. All parameters and offsets are initialized with randomly assigned nonzero regularization float. For cross-subject experiment on DEAP dataset, the total number of parameters in the whole model is about 50,156, which is larger than the total number of training samples. To prevent the overfitting of the model, a dropout layer is added after the first full connection layer of each BiLSTM, and the selected optimal dropout is 0.7.

## Experiments and Results

### Dataset and Preprocessing

To evaluate the proposed method, we make extensive experiments on the DEAP (Koelstra et al., 2012) dataset, which come from the Queen Mary University of London and is publicly and freely available for research on emotion recognition. This dataset records EEG, EMG, ECG, and other types of physiological signals induced by 32 subjects watching 40 music videos with different emotional tendencies. The emotion labels are evaluated with 1–9 consecutive values in four emotional dimensions of arousal, valence, preference, and dominance. In our research, we just take the EEG signals of each subject in 32 channels and 60 s from the DEAP dataset for study. The electrodes are positioned according to the 10–20 system. The sampling frequency is reduced to 128 Hz. For other artifacts, a 4- to 45-Hz bandpass filter is used for data filtering, and then blind source separation is used to remove the electro-oculogram (EOG) interference.

**FIGURE 3:**
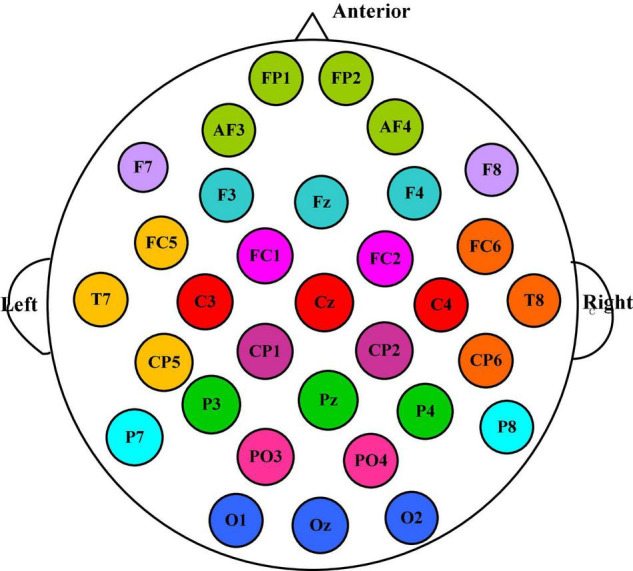
The 32 electrodes are divided into 12 clusters, among which the electrodes of the same color belong to the same brain area.

According to the spatial distribution of EEG electrodes, 32 electrodes are divided into 12 regions, that is, the number of brain regions *N* is 12, and each region contains at least two electrodes. We divided the 32 electrodes into 12 clusters or brain regions, where the electrodes of the same color belong to the same region, as shown in Figure 3. The electrodes contained in each brain region and the size of the corresponding manual feature set are listed in Table 1. In the DEAP database, there are 32 subjects, and each subject takes a 40-trial EEG data acquisition experiment. Each experiment collects 60 × 128 = 7,680 EEG records and emotional labels induced by watching videos for 60 s.

**TABLE 1:** Electroencephalogram electrodes and data size associated with each brain area.

Brain region	DEAP dataset		SEED dataset	
	Electrode name	Data size (d × n_*j*_)	Electrode name	Data size (d × n_*j*_)
Pre-frontal	FP1, FP2, AF3, AF4	4 × 4	AF3, FP1, FPZ, FP2, AF4	4 × 5
Frontal	F3, FZ, F4	4 × 3	F3, F1, FZ, F2, F4	4 × 5
Bilateral frontal	F7, F8	4 × 2	F7, F5, F6, F8	4 × 4
Left temporal	FC5, T7, CP5	4 × 3	FT7, FC5, T7, C5, TP7, CP5	4 × 6
Right temporal	FC6, T8, CP6	4 × 3	FT8, FC6, T8, C6, TP8, CP6	4 × 6
Frontal central	FC1, FC2	4 × 2	FC3, FC1, FCZ, FC2, FC4	4 × 5
Central	C3, CZ, C4	4 × 3	C3, C1, CZ, C2, C4	4 × 5
Central parietal	CP1, CP2	4 × 2	CP3, CP1, CPZ, CP2, CP4	4 × 5
Bilateral parietal	P7, P8	4 × 2	P7, P5, P6, P8	4 × 4
Parietal	P3, PZ, P4	4 × 3	P3, P1, PZ, P2, P4	4 × 5
Parietal occipital	PO3, PO4	4 × 2	PO5, PO3, POZ, PO4, PO6	4 × 5
Occipital	O1, OZ, O2	4 × 3	CB1, O1, OZ, O2, CB2	4 × 5

To balance the samples of three kinds of emotion labels in DEAP, the values 4 and 7 are used as the threshold to distinguish the positive, neutral, and negative emotion labels. As a result, for the total 32 subjects of the DEAP dataset, the number of positive, neutral, and negative trials are 373, 540, and 367, respectively. The proportion of samples in positive, neutral, and negative class is about 29%, 42%, and 29%, respectively. In this way, 40 trials were collected for each subject including 2,400-s EEG records, which is segmented according to 1 s, including 2,400 EEG segments. Each segment corresponds to three types of emotional labels: positive, neutral, and negative, of which there are about 800 segments of each type of emotional label. In this way, each subject has a total of 40 trials × 60 s = 2,400 s of EEG records, which were segmented by 1 s and contained a total of 2,400 EEG segments. Each segment corresponds to three types of emotions: positive, neutral, or negative tags. Then all segments are divided into 480 EEG samples according to T = 5. That means each sample contains five EEG segments, and DE features of four bands are extracted from 32 electrodes of each segment, so that each EEG sample is expressed by a manual feature tensor of 4 × 32 × 5, and the size of each EEG dataset is 480 × 4 × 32 × 5. The size of the 32-subject EEG dataset is 15,360 × 4 × 32 × 5.

To further prove the performance of our proposed model and make the conclusion more convincing, we also conducted serial comparison experiments on the SEED dataset (Zheng and Lu, 2017). The dataset collected EEG records related to emotional stimulation from 64 channels of 15 subjects (7 men and 8 women). The emotional labels fed back by the subjects were divided into positive, neutral, and negative. The dataset has been preprocessed, and DE features for each subject were extracted. On the SEED dataset, we also used trial-wise randomization method to construct cross validation sets for within-subject experiments and used the same leave-one-subject-out (LOSO) method as that used on the DEAP dataset to construct cross validation sets for cross-subject experiments. As for brain area division, to facilitate comparison, we removed the PO7 and PO8 electrodes and divided the remaining 62 electrodes into 12 brain areas. Table 1 shows the detailed brain area division method on the DEAP and SEED datasets.

### Benchmark Methods

For comparison, we use the following benchmark methods to perform within-subject and cross-subject emotion classification experiments on the same dataset.

The three traditional learning methods are the following: support vector machine (SVM) (Suykens and Vandewalle, 1999), bagging tree (BT) (Chuang et al., 2012), and random forest (RF) (Breiman, 2001).

The Seven deep learning methods are the following: deep confidence network (DBN) (Zheng and Lu, 2015), deep LSTM recurrent neural network (Alhagry et al., 2017), 2D-CNN (Chen et al., 2019c), 3D-CNN (Salama et al., 2018), hierarchical bidirectional GRU network based on attention mechanism (H-ATT-BGRU) (Chen et al., 2019b), domain adaptive neural network (DANN) (Ganin et al., 2016), and cascaded convolutional recurrent neural network (Casc-CNN-LSTM) (Chen et al.).

In order to horizontally compare the advantages of the proposed model, the input features of the benchmark models are also DE features extracted from four bands of EEG data in the DEAP and SEED datasets, which are consistent with those of our proposed model. The feature extraction method is the same as that stated in the experiment part of section “Within-Subject Experiment of Electroencephalogram Emotion Recognition.” *Classifier and discriminator*. However, the specific format of the input EEG features needs to be reshaped according to the interface of each model. Some key implementation details of these 10 benchmark models are listed in Table 2. The selection of model hyperparameters is also the result of fine-tuning experiments in the same search space mentioned in section “Discussion About Several Variants of the Proposed Model.*” Configuration and training of the bidirectional long- and short-term memory neural network from region to global brain model* of part II.

**TABLE 2:** Implementation details of 10 benchmark models.

Benchmark models	Input data size		Implementation details
	DEAP dataset	SEED dataset	
Support vector machine (SVM) (Suykens and Vandewalle, 1999)	[32 × 5, sample_size]	[Features, sample_size]	Kernel = ‘rbf’, gamma = 8, c = 0.05
Bagging tree (BT) (Chuang et al., 2012)	[32 × 5, sample_size]	[64 × 5, sample_size]	Method: bag, nLearn:100, weak learner: tree, type: classification
Random forest (RF) (Breiman, 2001)	[32 × 5, sample_size]	[64 × 5, sample_size]	n_estimators = 50, max_depth = 10, max_features = 8, min_samples_split = 20, min_samples_leaf = 10, oob_score = true, random_sate = 10
Deep confidence network (DBN) (Zheng and Lu, 2015)	[Batch_size, feature_size]: [60, 32 × 5]	[Batch_size, feature_size]: [60, 64 × 5]	hidden_layers = 3, hidden_size = 64, batch_size = 60, learning_rate = 0.04, dropout = 0.5, epochs = 200
Long- and short-term memory (LSTM) (Alhagry et al., 2017)	[Batch_size, seq_len, channels]: [120, 5, 32]	[Batch_size, seq_len, channels]: [120, 5, 64]	hidden_layers = 2, hidden_size = 64, seq_len = 5, batch_size = 120, learning_rate = 0.03, dropout = 0.5, num_directions = 2, epochs = 100 ×
Two-dimensional convolutional neural network (2D-CNN) (Chen et al., 2019c)	[Batch_size, seq_len × band_size, channels]: [60, 5 × 4, 32]	[Batch_size, seq_len × band_size, channels]: [60, 5 × 4, 64]	conv_layers = 2, max_pool_layers = 2, full_conn_layers = 2 (hidden_size = 128), conv_kernels = [32, 64], kernel_zize = [5 × 5, 3 × 3], pool_size = (2,2), batch_size = 60, learning_rate = 0.05, dropout = 0.7, epochs = 300, padding = 0, stride = 1
Three-dimensional convolutional neural network (3D-CNN) (Salama et al., 2018)	[Batch_size, band_size, seq_len, channels]: [80, 4, 5, 32]	[Batch_size, band_size, seq_len, channels]: [80, 4, 5, 64]	conv_layers = 2, max_pool_layers = 1, full_conn_layers = 1 (hidden_size = 128), conv_kernels = [8, 16], kernel_zize = [3 × 3 × 7, 2 × 2 × 5], pool_size = (2,2), batch_size = 80, learning_rate = 0.01, dropout = 0.6, epochs = 200, padding = 0, stride = 1
Hierarchical bidirectional GRU network based on attention mechanism (H-ATT-BGRU) (Chen et al., 2019b)	[Batch_size, band_size, seq_len, channels]: [60, 4, 5, 32]	[Batch_size, band_size, seq_len, channels]: [60, 4, 5, 64]	hidden_layers = 2, hidden_size = 64, seq_len = 5, batch_size = 60, learning_rate = 0.06, dropout = 0.4, num_directions = 2, epochs = 400
Domain adaptive neural network (DANN) (Ganin et al., 2016)	[Batch_size, feature_size]: [30, 32 × 5]	[Batch_size, feature_size]: [30, 64 × 5]	hidden_layers = 2, hidden_size = 128, batch_size = 30, L2-weight-regularization = 0.003, learning_rate = 0.02, dropout = 0.5, epochs = 500, momentum = 0.05, MMD regularization constant γ = 10e3
convolutional recurrent neural network (Casc-CNN-LSTM) (Chen et al., 2020)	[Batch_size, seq_len × band_size, channels]: [80, 5 × 4, 32]	[Batch_size, seq_len × band_size, channels]: [80, 5 × 4, 64]	CNN: conv_layers = 3, max_pool_layers = 3, full_conn_layers = 2 (hidden_size = 256), conv_kernels = [32, 64, 128], kernel_zize = [3 × 3, 3 × 3, 3 × 3], pool_size = (2,2), batch_size = 80, learning_rate = 0.05, dropout = 0.5, epochs = 500, padding = 0, stride = 1 LSTM: hidden_layers = 2, hidden_size = 128, seq_len = 256, batch_size = 80, learning_rate = 0.05, dropout = 0.5, num_directions = 2, epochs = 500

### Within-Subject Experiment of Electroencephalogram Emotion Recognition

**FIGURE 4:**
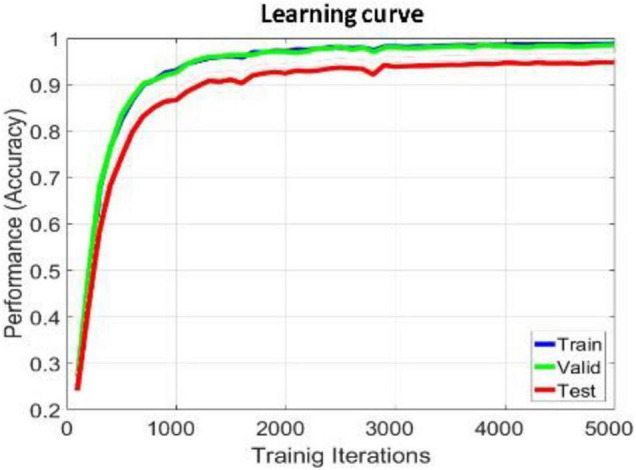
Learning process of R2G-ST-BiLSTM model in within-subject experiment on DEAP dataset.

We apply within-subject EEG emotion recognition method like that in literature (Li et al., 2018a) to evaluate our proposed model. To make the experiment result convincing, we use trial-wise randomization to construct the validation dataset. Specifically, we first picked out the subjects with a relatively balanced number of three types of trials. These 13 selected subjects include sub05, sub10, sub12, sub13, sub15, sub21, sub22, sub24, sub25, sub26, sub28, sub29, sub32. For each of these selected subjects, we randomly selected all segments of about 10% of the trials from each type as the test set, then randomly selected all segments of about another 10% of the trials from the rest of each type as the validation set, and at last take all segments of the remaining 80% of the trials as the training set. In this division process, we will make sure all segments belonging to one trial is allocated either as the training set, test set, or validation set to avoid “data leakage.” Then the proposed R2G-ST-BiLSTM model is used for feature learning and emotion classification. The learning process on the DEAP dataset is shown in Figure 4.

We use the average classification accuracy (ACC) and standard deviation (STD) of all subjects to evaluate the model performance. For comparison, we also use the abovementioned benchmark methods to make experiments on the equal dataset. We use paired t-test against the benchmark methods to show the difference between them. *T*-test is a test method for the difference between two mean values of small samples (sample size less than 30). It uses t-distribution theory to infer the probability of difference, to judge whether the difference is significant. The significance of the classification performance of the proposed method against each benchmark method is calculated with paired *t*-test. For all the paired *t*-tests, we used Bonferroni criteria (Genovese et al., 2002) and the implementation method (Weisstein, 2004) to make p-value correction for multiple hypothesis testing to limit false discovery rate (FDR). The results of within-subject experiment on the DEAP and SEED datasets are shown, respectively, in Tables 3, 4. The *p-*Value indicates the corrected results of paired *t*-test. A value of *p* < 0.05 means the difference is significant.

**TABLE 3:** The results of within-subject experiment on the DEAP dataset.

Method	SVM (Suykens and Vandewalle, 1999)	BT (Chuang et al., 2012)	RF (Breiman, 2001)	DBN (Zheng and Lu, 2015)	LSTM (Alhagry et al., 2017)	2D-CNN (Chen et al., 2019c)
Average classification accuracy (ACC)(%)/standard deviation (STD)	80.72/7.67	84.65/8.93	78.87/11.32	82.83/9.54	84.51/10.06	85.63/8.72
*p-*Value	0.0005	0.0004	0.0006	0.0008	0.0023	0.0019
Method	3D-CNN (Salama et al., 2018)	H-ATT-BGRU (Chen et al., 2019b)	DANN (Ganin et al., 2016)	Casc-CNN-LSTM (Chen et al., 2020)	R2G-ST-BiLSTM	
ACC (%)/STD	87.21/10.57	87.89/8.94	88.54/9.26	93.95/7.88	94.69/9.81	
*p-*Value	0.0052	0.0066	0.0074	0.0089		

**TABLE 4:** The results of within-subject experiment on the SEED dataset.

Method	SVM (Suykens and Vandewalle, 1999)	BT (Chuang et al., 2012)	RF (Breiman, 2001)	DBN (Zheng and Lu, 2015)	LSTM (Alhagry et al., 2017)	2D-CNN (Chen et al., 2019c)
ACC (%)/STD	80.14/9.27	83.72/8.68	77.95/9.32	82.58/11.26	83.92/9.44	84.64/7.98
*p-*Value	0.0006	0.0002	0.0004	0.0007	0.0009	0.0008
Method	3D-CNN (Salama et al., 2018)	H-ATT-GRU (Chen et al., 2019b)	DANN (Ganin et al., 2016)	Casc-CNN-LSTM (Chen et al., 2020)	R2G-ST-BiLSTM	
ACC/STD	87.31/11.14	86.38/9.56	88.96/10.45	92.72/9.33	93.57/8.52	
*p-*Value	0.0021	0.0035	0.0052	0.0098		

It can be seen from Tables 3, 4 that the average accuracy of the R2G-ST-BiLSTM method achieves 94.69% on DEAP and 93.57% on SEED, which is best among the above methods. From a statistical point of view, the performance of the proposed model is significantly better than the benchmark models. This result is largely because our R2G-ST-BiLSTM model explores both temporal and spatial context information of the different brain regions of EEG signals.

**FIGURE 5:**
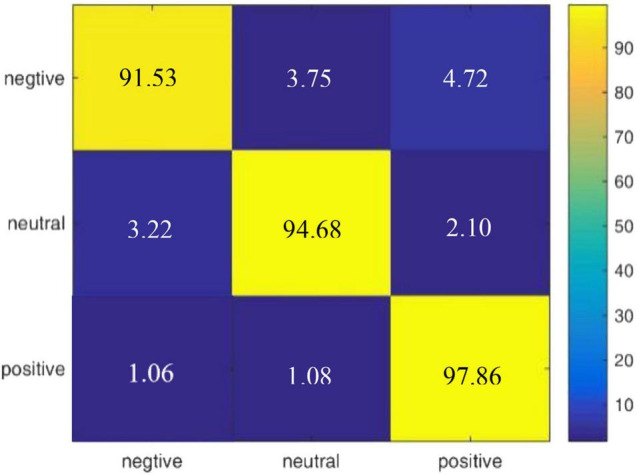
Confusion matrix of R2G-ST-BiLSTM model for within-subject experiment on DEAP dataset.

According to the experimental result of our proposed model on DEAP, we draw a confusion matrix for the three categories of emotions in Figure 5. It is found that compared with neutral emotions, positive and negative affections are less likely to be confused.

We also used a method-like reference (Zheng, 2017) to conduct some additional experiments to test the classification performance of different frequency bands of EEG data. Specifically, the DE features are extracted from four frequency bands θ, α, β, and γ related to the original signal, and then the EEG emotion recognition experiment is performed based on these DE features of the four bands. We can see the experimental results on DEAP and SEED datasets in Table 5, which indicate that on both datasets, the recognition performance in the higher frequency bands of β and γ is better than those in the lower frequency bands of θ and α. This result is consistent with the neurophysiology research in literature (Mauss and Robinson, 2009).

**TABLE 5:** The results of four frequency bands in within-subject experiment.

Methods	The results of ACC (%) (STD)							
	DEAP				SEED			
	θ	α	β	γ	θ	α	β	γ
SVM (Suykens and Vandewalle, 1999)	60.90 (8.76)	62.16 (10.49)	72.75 (7.87)	74.28 (11.13)	57.64 (9.93)	63.19 (7.55)	76.85 (10.19)	72.26 (12.31)
BT (Chuang et al., 2012)	65.44 (7.82)	67.31 (9.65)	77.52 (10.04)	76.06 (8.28)	61.65 (11.42)	62.74 (8.16)	75.58 (9.37)	78.83 (6.96)
RF (Breiman, 2001)	62.38 (12.52)	64.54 (7.39)	72.06 (9.77)	71.87 (10.45)	62.79 (7.58)	62.85 (12.32)	69.10 (11.14)	71.72 (10.56)
DBN (Zheng and Lu, 2015)	61.19 (8.97)	62.74 (11.25)	73.73 (6.80)	75.05 (7.74)	58.32 (9.44)	62.56 (10.28)	70.47 (13.51)	74.29 (8.84)
LSTM (Alhagry et al., 2017)	64.98 (9.15)	77.66 (10.57)	79.13 (7.22)	80.29 (8.83)	60.57 (11.89)	70.14 (12.67)	76.35 (10.23)	78.81 (9.50)
2D-CNN (Chen et al., 2019c)	65.73 (8.89)	68.45 (6.35)	79.96 (9.84)	81.42 (10.77)	67.22 (6.73)	69.36 (8.65)	77.24 (11.38)	80.58 (12.72)
3D-CNN (Salama et al., 2018)	65.26 (7.34)	70.17 (9.89)	82.51 (11.43)	83.68 (12.05)	64.54 (7.42)	71.09 (12.16)	78.67 (9.93)	82.11 (10.64)
H-ATT-BiGRU (Chen et al., 2019b)	66.27 (8.11)	68.58 (6.92)	81.96 (11.15)	84.25 (9.32)	65.03 (9.19)	67.15 (11.54)	81.58 (10.26)	85.34 (8.81)
DANN (Ganin et al., 2016)	68.39 (12.56)	70.87 (10.75)	85.73 (11.62)	86.92 (9.19)	67.56 (11.04)	72.42 (7.75)	79.96 (8.42)	85.47 (9.73)
Casc-CNN-LSTM (Chen et al., 2020)	70.07 (7.44)	73.25 (8.81)	88.54 (9.69)	89.18 (11.23)	69.21 (8.12)	75.88 (9.93)	85.25 (10.36)	89.53 (7.39)
R2G-ST-BiLSTM	**71.46 (10.73)**	**75.82 (9.55)**	**90.57 (7.36)**	**91.38 (8.92)**	**71.35 (8.28)**	**87.14 (6.67)**	**86.72 (9.81)**	**90.86 (11.92)**

*Bold values represent the better results obtained by the proposed method, highlighting the comparison.*

### Cross-Subject Experiment of Electroencephalogram Emotion Recognition

**FIGURE 6:**
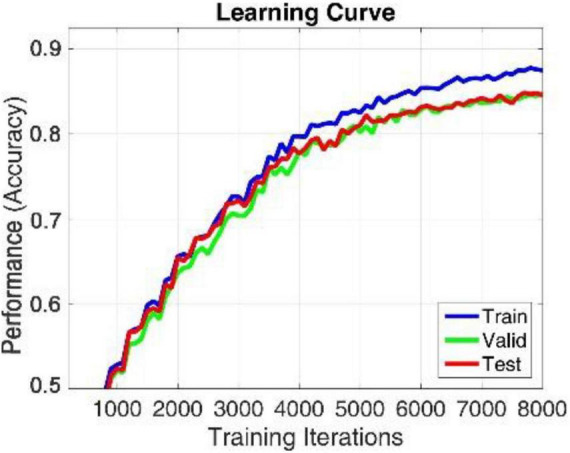
Learning process of R2G-ST-BiLSTM model in cross-subject experiment on DEAP dataset.

In this section, we use the cross-subject and the leave-one-subject-out (LOSO) cross-validation strategy similar to that in Zheng and Lu (2016); Li et al. (2018a) to evaluate the proposed method, in which the training and testing data are selected from different subjects. The EEG data of one subject is selected as test data, and the EEG data of all the rest of the subjects are used as training data. After each subject is rounded, the average prediction accuracy and standard deviation are calculated as the results. To better compare the performance of the proposed method, we use the abovementioned methods as benchmark. The comparison results of various methods on DEAP and SEED are illustrated in Tables 6, 7, respectively. On both datasets, our R2G-ST-BiLSTM model also performs better. The learning process on the DEAP dataset is shown in Figure 6.

**TABLE 6:** The results of cross-subject experiment on the DEAP dataset.

Method	SVM (Suykens and Vandewalle, 1999)	BT (Chuang et al., 2012)	RF (Breiman, 2001)	DBN (Zheng and Lu, 2015)	LSTM (Alhagry et al., 2017)	2D-CNN (Chen et al., 2019c)
ACC/STD	56.32/10.25	58.49/8.76	51.74/11.13	59.01/7.88	64.66/11.40	65.25/9.37
Method	3D-CNN (Salama et al., 2018)	H-ATT-BGRU (Chen et al., 2019b)	DANN (Ganin et al., 2016)	Casc-CNN-LSTM (Chen et al., 2020)	R2G-ST-BiLSTM	
ACC/STD	68.13/14.07	77.82/10.12	75.24/8.59	82.36/7.15	84.51/9.26	

**TABLE 7:** The results of cross-subject experiment on the SEED dataset.

Method	CM (Suykens and Vandewalle, 1999)	BT (Chuang et al., 2012)	RF (Breiman, 2001)	DBN (Zheng and Lu, 2015)	LSTM (Alhagry et al., 2017)	2D-CNN (Chen et al., 2019c)
ACC/STD	56.73/16.29	51.23/14.82	69.00/10.89	61.28/14.62	63.54/15.47	71.31/14.09
Method	3D-CNN (Salama et al., 2018)	H-ATT-BGRU (Chen et al., 2019b)	DANN (Ganin et al., 2016)	Casc-CNN-LSTM (Chen et al., 2020)	R2G-ST-BiLSTM	
ACC/STD	69.13/13.07	76.31/15.89	79.95/9.02	83.28/9.60	85.49/7.96	

**FIGURE 7:**
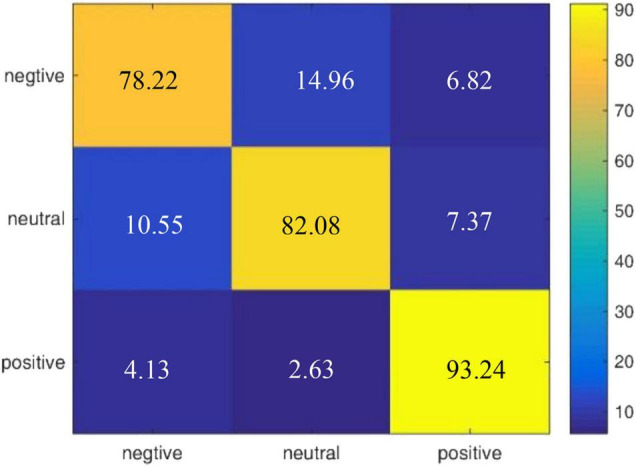
Confusion matrix of R2G-ST-BiLSTM model for cross-subject experiment on DEAP dataset.

We also draw a confusion matrix in Figure 7 according to the results of our model on the DEAP dataset, which shows that positive emotion is easier to be recognized than the negative and neutral emotions.

We also compared the influence of the different frequency bands on cross-subject emotion recognition. The experimental results on DEAP and SEED datasets are shown in Table 8, from which it can be seen that, on both datasets, the classification performance in the higher frequency bands of β and γ are better than those in the lower frequency bands of θ and α, and the R2G-ST-BiLSTM method achieves the best performance on the four frequency bands.

**TABLE 8:** The results of four frequency bands in cross-subject experiment.

Methods	The results (%) of ACC (STD)							
	DEAP				SEED			
	θ	α	β	γ	θ	α	β	γ
SVM (Suykens and Vandewalle, 1999)	41.91 (8.39)	44.73 (7.56)	48.66 (10.21)	51.32 (9.08)	40.62 (9.83)	42.05 (12.65)	47.97 (12.47)	50.06 (10.48)
BT (Chuang et al., 2012)	45.17 (9.38)	48.29 (14.77)	53.95 (8.54)	54.48 (7.43)	45.98 (9.70)	48.63 (10.28)	49.79 (12.41)	54.07 (6.87)
RF (Breiman, 2001)	41.50 (8.57)	41.86 (4.52)	47.31 (12.02)	47.72 (10.05)	40.07 (6.50)	42.09 (13.34)	48.29 (12.77)	48.98 (12.82)
DBN (Zheng and Lu, 2015)	44.36 (11.82)	46.15 (8.98)	55.94 (6.01)	56.81 (9.27)	45.76 (10.98)	48.43 (9.75)	56.66 (6.58)	56.62 (6.84)
LSTM (Alhagry et al., 2017)	47.92 (6.45)	48.69 (10.40)	59.02 (7.83)	59.16 (11.62)	48.63 (10.29)	51.59 (11.83)	62.13 (7.73)	59.37 (10.75)
2D-CNN (Chen et al., 2019c)	48.33 (7.61)	49.74 (13.26)	62.18 (9.90)	62.09 (11.31)	48.36 (10.31)	50.60 (8.30)	62.04 (6.74)	62.19 (7.62)
3D-CNN (Salama et al., 2018)	51.81 (9.79)	53.46 (9.84)	65.15 (11.32)	64.97 (8.46)	52.60 (11.84)	54.95 (10.45)	64.47 (13.69)	64.47 (14.69)
H-ATT-BiGRU (Chen et al., 2019b)	63.44 (12.50)	61.52 (7.07)	70.39 (12.14)	72.63 (5.28)	64.47 (14.96)	59,81 (12.43)	71.03 (10.48)	73.55 (8.80)
DANN (Ganin et al., 2016)	56.98 (5.33)	58.06 (11.80)	67.70 (8.65)	70.46 (12.17)	55.47 (9.80)	56.72 (10.79)	67.14 (7.17)	71.03 (10.14)
Casc-CNN-LSTM (Chen et al., 2020)	61.27 (8.02)	62.83 (6.56)	73.59 (10.54)	73.55 (8.69)	62.04 (6.64)	63.31 (11.96)	73.25 (9,12)	74.29 (7.98)
R2G-ST-BiLSTM	**64.03 (14.41)**	**66.26 (5.99)**	**74.64 (9.38)**	**75.02 (10.10)**	**66.14 (8.10)**	**67.14 (7.05)**	**74.85 (8.02)**	**75.89 (8.15)**

*Bold values represent the better results obtained by the proposed method, highlighting the comparison.*

**FIGURE 8:**
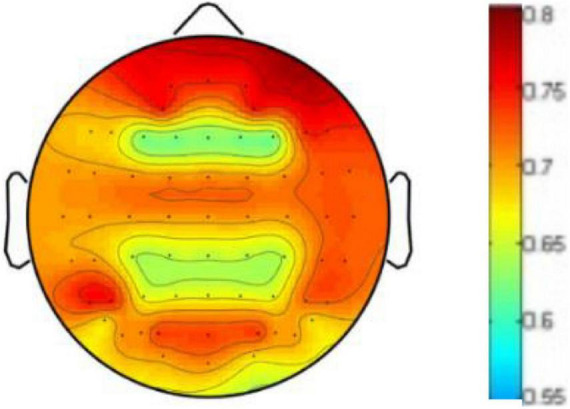
Weighted map of brain areas.

To prove the influence of the different brain regions on emotion recognition, we visualize the weight distribution of brain regions based on the weighting matrix W defined in formula (5) and learned in our cross-subject experiment on DEAP, where the sum of each row of W matrix represents the contribution of corresponding brain region. Figure 8 shows the weighted map of the brain areas, where the darker the color of the region, the more significant contribution of the corresponding brain region. It can be seen from Figure 6 that EEG signals in the frontal lobe are very important for human emotion recognition, which is consistent with the results of the cognitive observations of biological psychology in the literature (Coan and Allen, 2004).

### Discussion About Several Variants of the Proposed Model

Various experiments on the DEAP dataset demonstrates that the proposed R2G-ST-BILSM model is more effective than the other methods, which is largely due to our R2G-ST-BiLSM model utilizing both regional weighting layer and regional to global time layer. In order to confirm that, we obtained the following three simplified models by removing some layers from the R2G-ST-BiLSTM network, and use them to make within-subject and cross-subject experiments on the DEAP dataset. These three simplified models are described as follows:

(1) R2G-ST-BiLSTM-V1—removes the dynamic regional weighting layer and regional temporal feature learning layers;
(2) R2G-ST-BiLSTM-V2—only uses global temporal feature as the final input feature to classify;
(3) R2G-ST-BiLSTM-V3—does not change the original structure of the R2G-ST-BiLSTM, except that the weight of each brain region is set to 1, which means all brain regions are of the same importance to emotion classification.

Table 9 demonstrates the comparison outcome of the above four variant models. The comparison relationship is as follows:

**TABLE 9:** Comparison results of four models on the DEAP dataset.

Methods	Within-subject experiment	Cross-subject experiment
	ACC (%)	ACC (%)
R2G-ST-BiLSTM-V1	90.43	80.32
R2G-ST-BiLSTM-V2	91.58	81.15
R2G-ST-BiLSTM-V3	93.72	83.96
R2G-ST-BiLSTM	**94.69**	**84.51**

*Bold values represent the better results obtained by the proposed method, highlighting the comparison.*

R2G-ST-BiLSTM-V1 < R2G-ST-BiLSTM-V2 < R2G-ST-BiLSTM-V3 < R2G-ST-BiLSTM, (27)

The significance of the regional weighting layer and the regional temporal feature learning layer has been proven by the above comparisons, which shows that these two parts play important roles in enhancing the capability of our R2G-ST-BiLSTM model.

To further discuss whether the different components of R2G-ST-BiLSTM are necessary to outperform other models, we modified it according to the following methods to obtain its several variants:

(1) R2G-ST-CNN-V1: replaces all BiLSTM modules used for learning spatial and temporal features of local and global brain regions with two-layer 2D-CNN modules.
(2) R2G-ST-CNN-V2: only the BiLSTM modules used for learning temporal features of local and global brain regions are replaced with two-layer 2D-CNN modules.
(3) R2G-ST-CNN-V3: only the BiLSTM modules used for learning spatial features of local and global brain regions are replaced with two-layer 2D-CNN modules.
(4) R2G-ST-BiLSTM-V4: only remove the domain discriminator from the proposed model.

The structure and parameter configuration of the 2D-CNN here are consistent with those in literature (Chen et al., 2019c). These four variant models are used to make within-subject and cross-subject emotion classification experiments on DEAP. The comparison results are shown in Table 10.

**TABLE 10:** Comparison results of five models on the DEAP dataset.

Methods	Within-subject experiment	Cross-subject experiment
	ACC (%)	ACC (%)
R2G-ST-CNN-V1	85.26	75.64
R2G-ST-CNN-V2	88.75	80.72
R2G-ST-CNN-V3	90.83	81.47
R2G-ST-BiLSTM	**94.69**	**84.51**
R2G-ST-BiLSTM-V4	92.14	78.39

*Bold values represent the better results obtained by the proposed method, highlighting the comparison.*

It can be seen from Table 10 that the classification performance of the proposed model is significantly better than that of the four variant models. Specifically, the proposed model outperforms the R2G-ST-CNN-V2, which indicates that the BiLSTM components can extract more discriminative time-context features from EEG sequences than 2D-CNN. The performance of our proposed model is better than that of R2G-ST-CNN-V3, which shows that BiLSTM components can better cooperate with the attention mechanism of brain regions and extract more spatial context-dependent features than 2D-CNN. The proposed model significantly outperforms R2G-ST-CNN-V1, which further proves that BiLSTM has obvious advantages over 2D-CNN in learning deep temporal and spatial features in our proposed hierarchical framework. The proposed model significantly outperforms R2G-ST-CNN-V4, especially in cross-subject experiment, which illustrates that the domain discriminator is indeed helpful to extract more discriminative EEG features with small differences between subjects and, therefore, improve the adaptability of the model. In general, the components of BiLSTM and the domain discriminator play very important roles on the whole performance of the proposed model and are necessary to outperform other models.

## Discussion

Although the proposed model has achieved high classification accuracy, there are still some limitations to study and overcome in the future.

At first, the model is complex and lacks interpretability. The model proposed in this paper is a combined hierarchical deep neural network composed of multiple bidirectional LSTM models with attention mechanism. Although the principle and learning process of the model is clear, the decision making and intermediate process made by the model are difficult to understand and interpreted. It is hard to explain the correlation and the interaction among input data, learned features, and output class. At present, researchers have put forward some specific deep model interpretation methods including activation maximization, gradient-based interpretation, class activation mapping (CAM), and so on. The interpretation result of the activation maximization is more accurate and can help people understand the internal working logic of DNN, but the data containing some noise generated in the optimization process makes it difficult to interpret the input (Dong et al., 2017). The gradient-based interpretation methods include deconvolution, guided backpropagation, integrated gradients, and smooth gradients, which aim to use backpropagation to calculate the gradient of specific output relative to input to derive feature importance. This gradient information can only be used to locate important features, but not to quantify the contribution of each feature to the classification results. The CAM method (Jorg, 2019) can locate the objects from the learned features by the excellent ability of the last convolution layer in CNN, which could only provide coarse-grained interpretation results for various CNN models. Additionally, there are some model-agnostic (MA) explanations, such as LIME and knowledge distillation, and causal interpretable method. Although many methods have been proposed in the interpretability research for deep models, there are still many problems to be solved, such as the lack of unified indicators for evaluating interpretation methods, the balance between model accuracy and interpretability, and the balance between data privacy protection and model interpretability, which will be one of our future research directions to improve the performance of the model.

Second, the complex model and limited amount of data make the model prone to overfitting. We use the EEG data of the DEAP and SEED datasets, which include 32 and 15 subjects, respectively, to make our experiments. In cross-subject experiments on the DEAP dataset, the number of the model parameters reaches about 50,156, which exceeds the number of training samples at 15,360. Compared with the complexity of the model, the training dataset is small, which makes the model prone to overfitting. At present, researchers usually use methods such as expanding dataset, removing features, regularization, and terminating training in advance to prevent model overfitting (Sanjar et al., 2020). Data enhancement is a way to increase training data, which can be realized by flipping, translation, rotation, scaling, and generation methods. Removing features is to reduce the complexity of the model by removing some layers or some neurons from it. Through monitoring the performance of each training iteration and when the loss on the verification set tends to increase, we could stop the training process to prevent the model from overfitting. The regularization method reduces the complexity of the model by punishing the loss function with L1 or L2 paradigm. In our work, we use L2 regularization and dropout method to suppress the overfitting problem, but we still face the challenge of insufficient data. In the future, we will design experiments or ask some medical institutions or hospitals to collect more EEG data for the study. We will also explore to use the generated antagonism network (GAN) to generate a large number of artificial EEG data to make up for this deficiency.

Third, the proposed model is so complex that it needs to consume a lot of computation resources and time to train the model, and it is hard to quickly verify and improve the model, as well as make real-time prediction. In the future, we will try to further simplify the structure of the model without changing its performance, and make deep research on accelerating the speed of model training and real-time application.

## Conclusion

Based on the discovery of neuroscience that each region of the human brain can produce different dynamic responses to emotions, we suggest a new hierarchical EEG feature learning method by using attention mechanism and bidirectional LSTM neural network from region to global brain. A large number of experiments and verification are carried out on the DEAP and SEED datasets. The results show that the proposed R2G-ST-BiLSTM model achieves the best performance in subject-dependent and subject-independent EEG-based emotion recognition. Through experiments on several variants of the model, we compare and analyze the impact of different components of the model on its overall performance, and summarize the following advantages of the proposed model:

(1) The BiLSTM networks are used to hierarchically learn the deep spatial correlation features within and cross each brain region. The attention mechanism is combined to weigh the contribution of each brain region to the emotion classification, which could enhance the influence of the brain region with more contribution and reduce the influence of the brain region with less contribution.
(2) The BiLSTM networks are used to hierarchically learn the deep temporal correlation features from the EEG time sequence of each local brain region and global brain. The learned deep temporal and spatial features are connected to make the features more discriminative.
(3) By introducing the domain discriminator, the feature difference between different subjects is reduced, and the robustness and adaptability of the model are improved.

Although the proposed model shows some advantages, there are still some problems to be solved. For example, the model is more complicated, which costs much time and computing resource for training. The whole proposed model still works as a black box, and it is difficult to explain the physical meaning represented by the learned abstract features. The complex model and limited amount of data make the model prone to overfitting. Therefore, in the future, we will further study how to improve the interpretability of the proposed model, simplify the structure of the model, and further improve the robustness and domain adaptability of the model.

## Data Availability Statement

The original contributions presented in the study are included in the article/supplementary material, further inquiries can be directed to the corresponding author/s.

## Author Contributions

PZ designed and implemented the R2G-ST-BiLSTM model and participated in drafted the manuscript. CM carried out the cross-subject EEG-based emotion classification experiments with R2G-ST-BiLSTM model, analyzed the experimental results, and drafted the manuscript. KZ carried out the within-subject experiments and analyzed the experimental results. WX was responsible for literature review, EEG data preprocessing and manual feature extraction. JC conceived of the study, participated in its design, and revised and proofread the manuscript. All authors read and approved the final manuscript.

## Conflict of Interest

The authors declare that the research was conducted in the absence of any commercial or financial relationships that could be construed as a potential conflict of interest.

## Publisher’s Note

All claims expressed in this article are solely those of the authors and do not necessarily represent those of their affiliated organizations, or those of the publisher, the editors and the reviewers. Any product that may be evaluated in this article, or claim that may be made by its manufacturer, is not guaranteed or endorsed by the publisher.
